# Clinical and Genetic Features of a Large Monocentric Series of Familial Non-Medullary Thyroid Cancers

**DOI:** 10.3389/fendo.2020.589340

**Published:** 2021-01-07

**Authors:** Valentina Cirello, Carla Colombo, Olga Karapanou, Gabriele Pogliaghi, Luca Persani, Laura Fugazzola

**Affiliations:** ^1^ Department of Endocrine and Metabolic Diseases, Istituto di Ricovero e Cura a Carattere Scientifico (IRCCS) Istituto Auxologico Italiano, Milan, Italy; ^2^ Department of Pathophysiology and Transplantation, Università degli Studi di Milano, Milan, Italy; ^3^ Department of Endocrinology, 401 Military Hospital, Athens, Greece; ^4^ Department of Medical Biotechnology and Translational Medicine, University of Milan, Milan, Italy

**Keywords:** FNMTC, familial non-medullary thyroid cancer, DUOX2, familial thyroid cancer, outcome, HABP2, MAP2K5

## Abstract

Several low penetration susceptibility risk loci or genes have been proposed in recent years with a possible causative role for familial non-medullary thyroid cancer (FNMTC), though the results are still not conclusive or reliable. Among all the candidates, here fully reviewed, a new extremely rare germline variant c.3607A>G (p.Y1203H) of the *DUOX2* gene, has been recently reported to co-segregate with the affected members of one non-syndromic FNMTC family. We aimed to validate this finding in our series of 33 unrelated FNMTC Italian families, previously found to be negative for two susceptibility germline variants in the *HABP2* and *MAP2K5* genes. Unfortunately, the *DUOX2* p.Y1203H variant was not found in either the 74 affected or the 12 not affected family members of our series. We obtained interesting data by comparing the clinico-pathological data of the affected members of our kindreds with a large consecutive series of sporadic cases, followed at our site. We found that familial tumors had a statistically significant more aggressive presentation at diagnosis, though not resulting in a worst outcome. In conclusion, we report genetic and clinical data in a large series of FNMTC kindreds. Our families are negative for variants reported as likely causative, namely those lying in the *HABP2*, *MAP2K5* and *DUOX2* genes. The extensive review of the current knowledge on the genetic risk factors for non-syndromic FNMTCs underlies how the management of these tumors remains mainly clinical. Despite the more aggressive presentation of familial cases, an appropriate treatment leads to an outcome similar to that observed for sporadic cases.

## Introduction

The term familial non-medullary thyroid cancers (FNMTCs) is used to indicate thyroid tumors, arising from follicular cells, which are observed in two or more first-degree relatives in the absence of predisposing environmental factors. FNMTCs account for 3–9% of all thyroid cancer (TC) cases, and show an autosomal dominant pattern of inheritance with incomplete penetrance and variable expressivity (1). FNMTC is further sub-classified in: syndromic FNMTCs (5% of all FNMTCs) in which TC occurs as a minor component (familial adenomatous polyposis, Gardner’s syndrome, Cowden’s syndrome, Werner’s syndrome, McCune-Albright syndrome, Carney complex type 1, DICER1 syndrome) and non-syndromic FNMTCs (95% of all FNMTCs), in which TC is the predominant feature. Among FNMTCs, papillary thyroid cancer (PTC) is the commonest histological subtype (2, 3). Some studies reported that non-syndromic familial PTCs (FPTCs) are more aggressive than sporadic PTCs, in terms of higher rate of multifocal and bilateral tumors, extrathyroidal extension, lymph node metastasis, younger age of onset, and recurrence rate (4–8). Moreover, the phenomenon of “anticipation” with early and more severe manifestations of the disease has been observed in the second generation (9). On the contrary, other studies did not found clinical differences between non-syndromic FNMTCs and sporadic cases (10, 11). FNMTCs are indistinguishable from sporadic NMTC forms by either morphological examination or immunohistochemical staining, implying that FNMTCs cannot be diagnosed until at least one of the patient’s first-degree relatives is diagnosed with a thyroid cancer. Genetic alterations underlying syndromic forms have been at least in part defined (*APC* in familial adenomatous polyposis and Gardner’s syndrome; *PTEN* in Cowden’s syndrome; *PRKAR1A* in Carney Complex type 1; *WNR* in Werner’s syndrome, *DICER1* in DICER1 syndrome; *GNAS* in McCune Albright syndrome) (1, 3). On the other hand, the genetic susceptibility for non-syndromic FNMTCs is still not well defined. To date, only few susceptibility risk chromosomal loci [*MNG1* (14q32), *TCO* (19p13.2), *fPTC/PRN* (1q21), *NMTC1* (2q21), *FTEN* (8p23.1–p22), 6q22, 4q32, and 8q24] (1) and susceptibility genes (*TTF-1/NKX.2*, *SRGAP1*, *FOXE1*, *SRRM2*, *HABP2*, *MAP2K5*) (12–17) have been identified by means of linkage analysis, genome-wide association study (GWAS), whole exome (WES) and next-generation sequencing (**Table 1**). In addition, an imbalance of the telomere–telomerase complex has been reported in the peripheral blood of familial NMTC patients (9). Nevertheless, the reliability of these genetic alterations is controversial and still under investigation. Very recently, Bann and colleagues identified a novel germline variant, c.3607T>A (p.Y1203H) in *Dual Oxidase-2* (*DUOX2*) gene, segregating as an autosomal dominant trait in all affected individuals from a non-syndromic FNMTC family. This variant is extremely rare in the general population (prevalence of 1 out of 138,000 individuals reported in the dbSNP database), consistent with its role as a causative mutation in a rare condition such as FNMTC (34). *DUOX2* gene (MIM*606759) maps on chromosome 15 and encodes a protein comprising an extracellular N-terminal peroxidase-like domain, seven transmembrane domains, two cytosolic Calcium-binding sites (EF hand motifs), and an intracellular C-terminal oxidase domain. The maturation factors DUOXA1 and DUOXA2 are essential components for the enzymatic activity of DUOX2, because they form heterodimeric complexes with DUOX2 and allow the translocation of the dimer from endoplasmic reticulum to the membrane (35). The *DUOX2* p.Y1203H variant affects an amino acid highly conserved across mammalian, localized in the fifth transmembrane domain of DUOX2 protein. Due to its location, it is unlikely that the variant could impair the enzymatic active site of the protein or the interaction with DUOX2A.

**Table 1:** Susceptibility genes and variants identified in non-syndromic familial non-medullary thyroid cancer (FNMTC).

Gene (chr locus)	Variant	Pos affected members/tot (%) (pos FNMTC families/tot) Co-segregation	Pos sporadic NMTC/tot (%)	Pos healthy controls/tot (%)	Origin of population studied	Methodology/Functional study	References
**FOXE1 **(9q22.33)	A248G	3/145 (2.1%) ^£^ (1/60) Yes	1/80 (1.3%)	0/130	Portuguese	Sequencing Yes	Pereira (14)
**TTF-1 **(14q13.3)	A339V	7 ^¥^ (2/2) Yes	4/20 (20%) ^≠^ 0/284 €	0/349	Mostly Chinese	Sequencing Yes	Ngan (12)
		0/63 (0/38) -	–	–	Italian	Enzymatic digestion -	Cantara (18)
**SRGAP1 **(12q14.2)	Q149H	2 1/21 Yes	0/367^⌠^ 0/432^∫^	0/552^⌠^ 0/424^∫^	Caucasian non-Hispanic; Caucasian Hispanic	GWAS Yes	He (13)
	A275T	3 1/21 Yes					
**SRRM2 **(16p13.3)	S346F	6 ^∞^ (1/138) Yes	7/1,170 (0.6%) ^∞^	0/1,404	Caucasian, African American, Asian	Genotyping, WES, haplotype, and linkage analysis	Tomsic (15)
**HABP2 **(10q25.3)	G534E	7 (1) Yes	4.7% of 423 ^ø^	0.7%	Unknown	WES Yes	Gara (16)
		6/43 (14%) (4/29) Yes (1 family)	–	–	Unknown	Sequencing -	Zhang (19)
		0 (0/12) -	0/217	–	Chinese	Sequencing -	Zhao (20)
		0/11 (0/4) -	1/509 (0.2%)	1/190 (0.5%)	Middle Eastern	Sequencing -	Alzahrani (21)
		0/63 (0/38) -	–	–	Italian	DHPLC, sequencing -	Cantara (22)
		0/59 (0/16) -	–	–	Brazilian	Sequencing -	De Mello (23)
		11/179 (6.1%) (11/179) No (6 families) Yes (2 families)	93/1,160 (8%)	121/1,395 (8.7%)	Caucasian	SNaPshot assay -	Tomsic (24)
		2/33 (6.1%) (1/16) No	2/62 (3.2%)	6/267 (2.2%)	Spanish	Not specified -	Ruis-Ferrer (25)
		1/37 (2.7%) (1/37) No	–	351/4,634 (7.6%) ^#^ 111/1,195 (9.3%) ^@^	Australian	Sequencing -	Weeks (26)
		5/63 (7.9%) (3/27) No	–	–	Italian	Sequencing -	Colombo (27)
		–	171/2,095 (8.2%)	238/5,172 (9.1%)	British	KASP genotyping -	Sahasrabudhe (28)
		–	1.4% of 281	1.3% of 1,105	Hispanic	Competitive allele-specific genotyping -	Bohorquez (29)
		–	12/326 (3.7%)	19/400 (4.7%)	Polish	Sequencing -	Kowalik (30)
		2/20 (10%) (1/11) No	8/136 (5.9%)*	–	European	Sequencing, SnapShot -	Kern (31)
	R122W	3/20 (15%) (2/11) Yes	2/136 (1.4%)	–	European	Sequencing, SnapShot -	Kern (31)
		0/72 (0/32) -	–	–	Italian	Sequencing -	Colombo (32)
**MAP2K5 **(15q23)	A321T M367I	3/77 (3.9%) 2/77 (2.6%) (2/34) Yes	0/507 ^ø^	0.023% of 2,200 ^β^	Chinese	WES Yes	Ye (17)
	A321T M367I	0/74 0/74 (0/33) -	–	–	Italian	Sequencing -	Cirello (33)

£, a fourth member, harboring the variant and not included, had an ovaric cancer; ¥, both PTC/MNG and MNG; ≠, PTC/MNG; €, only PTC; ∞, Caucasian; ⌠, from Ohio; ∫, from Poland; ø, from TGCA database; α, from Multiethnic database; *, sporadic and familial MTC; #, from Busselton Health Study participants (unknown disease status); @, form TwinsUK participants (no history of thyroid cancer); β, from Novo-Zhonghua genomes; WES, whole exome sequencing.

In the present study, we aimed to validate the hypothesized role of this *DUOX2* p.Y1203H germline variant in our series of FPTC Italian families. We also carried out a review of literature in order to summarize the current knowledge on the genetic risk factors for FNMTCs. Finally, the clinical features and the outcome of our familial cases were compared with those of our consecutive series of sporadic PTC cases.

## Patients and Methods

### Patients

The presence of *DUOX2* p.Y1203H germline variant was evaluated in a series of 33 unrelated Italian FNMTC kindreds, followed-up in our tertiary center for thyroid cancer care during the period 2003–2019. All affected members had a papillary thyroid cancer (FPTC). By definition, all families had a PTC in first-degree relatives and, in particular, 12/33 (36%) families had a parent-child relationship, 19/33 (58%) had a sibling relationship and 2/33 (6%) families had an uncle-nephew relationship. The number of affected members was two in 27 families, three in 4 families, and four in 2 families. Families came from different regions of Italy, North, South, and islands. Tumors were classified and staged according to the thyroid malignancy World Health Organization classification and the 8th edition of TNM staging (36). Patients were diagnosed and treated according to the recent guidelines for the management of thyroid cancer (37, 38). To identify remission or persistent/recurrent disease, ATA guidelines and Italian consensus recommendations were followed (37, 38). Clinico-pathological features at diagnosis and the final outcome, after a mean follow-up of 93 months, were available for all FPTC probands and for additional 10 affected members. None of kindreds analyzed had clinical features consistent with a possible familial cancer syndrome and none of the family members reported had a history of other primary cancers, except one member who was affected with a tongue carcinoma in remission. The clinical data of the FPTC patients were compared with those coming from all the sporadic PTCs (n = 1,186) included in the database of our tertiary Center. An Institutional Review Board approval was obtained for the analysis of the clinical and molecular data, and informed consent was given by all screened subjects.

### Genotyping

Germline DNA was extracted from the peripheral blood of 86 family members (74 affected with FPTC and 12 unaffected), using standard methods. The exon 28 of the *DUOX2* gene, where the genetic variant c.3607A>G (p.Y1203H) lies, was amplified by PCR and sequenced on ABI PRISM 3100 automated genetic analyzer (Applied Biosystems, Waltham MA, USA) both in forward and reverse directions, as previously reported (39). A subset of 14 thyroid tumor samples from our kindreds were analyzed using the custom PTC-MA assay, based on matrix-assisted laser desorption/ionization time-of-flight mass spectrometry, and previously set up for the simultaneous identification of 13 known hotspot mutations *BRAF* ^V600E^; *AKT* ^1E17K^; *EIF1AX* c.338-1G>C; *NRAS* ^Q61R^ and *NRAS* ^Q61K^; *HRAS* ^G13C^, *HRAS* ^Q61K^, and *HRAS* ^Q61R^; *KRAS* ^G12V^ and *KRAS* ^G13C^; *TERT* c.-124C>T and *TERT* c.-146C>T; and *PIK3CA* ^E542K^ and 7 recurrent fusion genes typical of PTC: *RET/PTC1* (RET/CCDC6), *RET/PTC2* (RET/PRKAR1A), and *RET/PTC3* (RET/NCOA4) and *TRK* (NTRK1/TPM3), *TRK-T1* (NTRK-T1/TPR), and *TRK-T3* (NTRK1/TFG) (40, 41).

### Statistical Analysis

Relations between discrete variables were evaluated by means of t-test or chi^2^ test, as appropriate. Statistical significance was defined as P <0.05. All statistical analyses were performed using MedCalc Analyses (Version 18.11.3 of the MedCalc Software, B-8400 Ostend, Belgium).

## Results

### Clinico-Pathological and Molecular Features

Clinical data were available for all probands and for 10 additional affected family members, for a total of 43 patients (38 females and 5 males), with a median age at diagnosis of 44 years (**Table 2**). In particular, most patients underwent surgery due to a malignant (67%) or suspicious (5%) cytology, while thyroid carcinoma evidence was incidental in 28% of cases. Mean tumor diameter was 13.9 mm, and the prevalence of papillary microcarcinomas was of 37%. Moreover, the prevalence of multifocal carcinomas, unilateral or bilateral, was 56%, and the prevalent variant of papillary histotype was the classical one (81%). Gross extrathyroidal extension, defined by the 8th edition of TNM staging as the presence of macroscopic tumor extending outside the thyroid gland, was recorded in 35% of cases, while metastatic lymph nodes and lung metastases were found in 26 and 7% of cases, respectively.

**Table 2:** Clinico-pathological features at diagnosis of 43 patients with familial papillary thyroid cancer (FPTC).

Features	n (%)
Median age at diagnosis, range	44 years, 8–81
Female/male gender	38/5 (88/12)
Pre-surgical diagnosis yes/indeterminate/no	29/2/12 (67/5/28)
Total thyroidectomy/lobectomy	40/3 (93/7)
Lymph-nodes dissection yes/no	18/25 (42/58)
Mean tumor size, range	13.9 mm, 1.5–90
Tumor size ≤10 mm/>10 mm	16/27(37/63)
Histological variants of PTC (classical/follicular/other)	35/7/1 (81/16/3)
Gross extrathyroidal extension yes/no	15/28 (35/65)
Multifocality yes/no	24/19 (56/44)
T1/T2/T3/T4	28/3/9/3 (65/7/21/7)
N1/N0/NX	11/7/25 (26/16/58)
M0/M1	40/3 (93/7)
AJCC Stage (I/II/III/IV)	38/0/1/4 (88/0/3/9)
Radioiodine ablation yes/no	27/16 (63/37)
Remission/persistence	34/9 (79/21)
Mean follow-up, range	92.2 months, 12–238

AJCC, American Joint Committee on Cancer.

None of the clinico-pathological parameters analyzed were found to be significantly different among the 27 families with 2 affected members and the 6 families with 3 or more affected members (data not shown).

**Figure 1:**
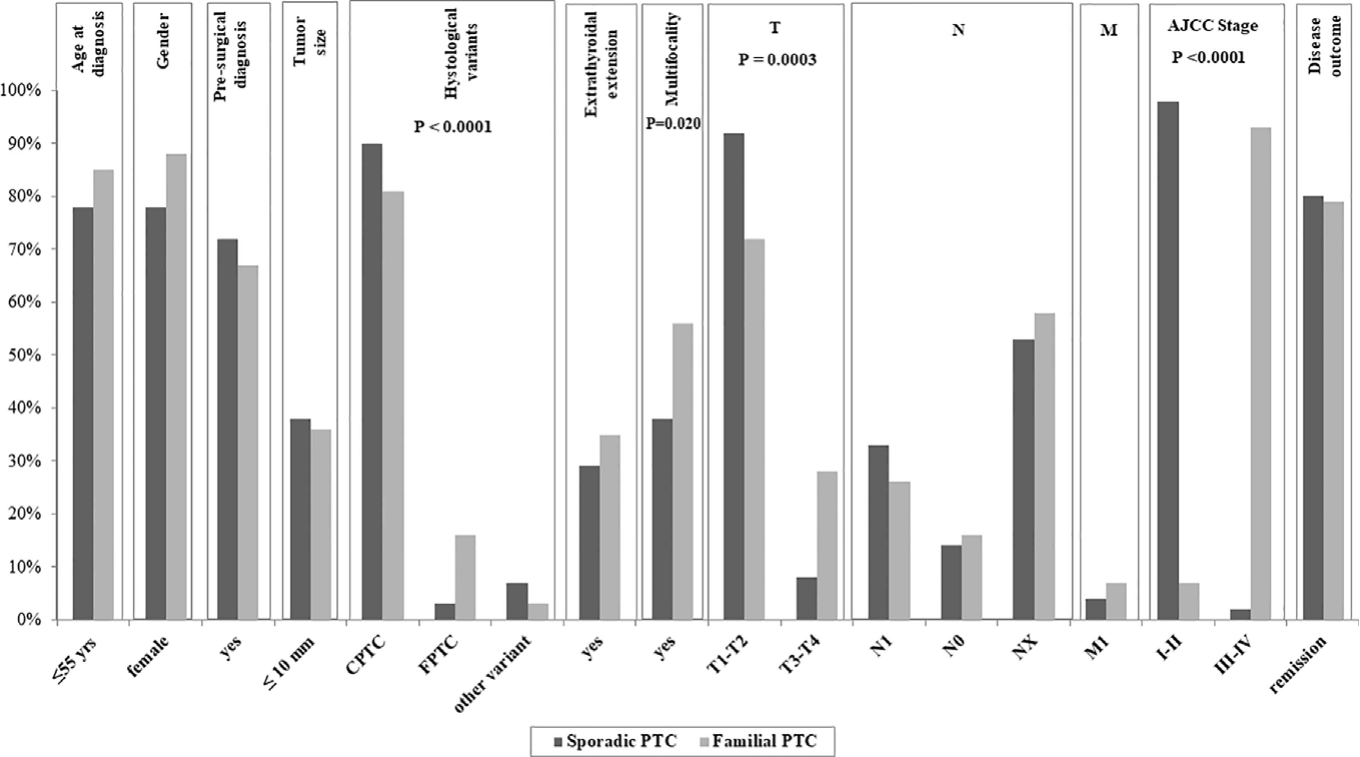
Clinico-pathological data of the affected members of our Familial Papillary Thyroid Cancer (FPTC) kindreds compared with those of the sporadic PTC cases followed at our site. The P value is reported only for the features with significant differences between familial and sporadic cases.

Clinical data related to the 1186 sporadic PTC followed at our site are reported in **Table 3**. Interestingly, statistically significant differences were found between FPTC and sporadic PTC cases (**Figure 1**), with the former presenting with worse prognostic features. In particular, in FPTCs the histological variants other than the classical one were more represented (19 vs 10%, P < 0.0001),

**Table 3:** Clinico-pathological features of 1,186 sporadic papillary thyroid cancer patients at diagnosis.

Features	n, %
Median age at diagnosis, range	44 years, 7–87
Female/male gender	921/265 (78/22)
Pre-surgical diagnosis yes/indeterminate/no	722/70/394 (61/6/33)
Total thyroidectomy/lobectomy	1,142/44 (96/4)
Lymph-nodes dissection yes/no	559/627 (47/53)
Mean tumor size, range	16 mm, 1–90
Tumors ≤10 mm/>10 mm	451/735 (38/62)
Histological variants of PTC (classical/follicular/other)	1,063/36/87 (90/3/7)
Gross extrathyroidal extension yes/no	346/840 (29/71)
Multifocality yes/no	454/732 (38/62)
T1/T2/T3/T4	932/151/75/28 (79/13/6/2)
N1/N0/NX	397/162/627 (33/14/53)
M0/M1	1,144/42 (96/4)
AJCC Stage (I/II/III/IV)	1,100/76/7/3 (92/6/1/1)
Radioiodine ablation yes/no	725/461 (61/39)
Remission/persistence	952/234 (80/20)
Mean follow-up, range	100 months, 6–533

AJCC, American Joint Committee on Cancer.

multifocality was significantly more frequent (56 vs 38%, P = 0.02), T3–T4 tumors were more prevalent (28 vs 8%, P = 0.0003), as well as the AJCC III-IV stage at diagnosis (12 *vs* 2%, P < 0.0001). Other clinico-pathological features did not differ between the two groups, namely age at diagnosis, gender, tumor size, pre-surgical diagnosis, gross extrathyroidal extension, local and distant metastases, and disease outcome (**Figure 1**).

Moreover, the prevalence of patients undergoing radioiodine therapy was similar between two PTC groups (63% in FPTC *vs* 61% in sporadic PTC, P = 0.826), as well as the mean follow-up (92.2 *vs* 100 months, respectively, P = 0.587).

Among the 33 families studied, 74 patients with FPTC and 12 healthy subjects were submitted to genetic analyses. The molecular analysis of *DUOX2* exon 28 did not show the presence of the p.Y1203H germline variant in any affected or healthy family member.

Interestingly, the genetic characterization of the 14 FPTCs, for whom the tumor tissue was available, revealed a *BRAFV600E* mutation in 4/14 (28.6%) cases, a *RET* fusion in 2/14 (14.3%) (1 with *ret/PTC1* and 1 *ret/PTC3*), a *TERT* c.-124C>T mutation in 2/14 (14.3%), while 1/14 (7.1%) tumor harbored both *NRAS*Q61K and *TERT* c.-146C>T mutations, and 1/14 both *BRAF*V600E and *TERT* c.-124C>T genetic variations. Finally, 4/14 (28.6%) cases were wild type for the mutations and fusions tested. Of note, 9/10 patients harboring somatic mutations owned to families with two affected members, while only 1 patient belonged to a family with three affected members.

## Discussion

The genetic susceptibility for non-syndromic FNMTCs is still not well defined, with few susceptibility risk chromosomal loci (1) and susceptibility genes (12–17, 31) identified whose reliability is controversial and still under investigation.

In the present study, our large series of Italian unrelated FNMTC kindreds, in which we previously excluded a role for *HABP2* and *MAP2K5* germline variants (27, 32, 33), has been found not to harbor the *DUOX2* p.Y1203H variant, recently described in a non-syndromic FNMTC family (34). Mutations in the *DUOX2* gene, whose protein function is to generate hydrogen peroxidase required for thyroid hormone synthesis in the follicular cells, are involved in the development of congenital hypothyroidism, and are typically found in the N-terminal extra cellular peroxidase-like domain and in the EF hand domains, where they compromise the enzymatic activity of the protein. On the contrary, only few mutations have been described in transmembrane domains (35). The p.Y1203H variant has a transmembrane localization and the derived mutant protein could be mislocalized, thus resulting in the dysregulation of H_2_O_2_ metabolism. Thus, since hydrogen peroxidase could be involved in tumorigenesis through oxidative DNA stress, cell signaling and proliferation, regulation of gene expression, and cell senescence and apoptosis (42), the variant was considered of potential interest. Moreover, functional studies demonstrated that the mutated DUOX2 protein is functionally active and capable of producing H_2_O_2_, with an increase production rate of reactive oxygen species (ROS) at plasma membrane level than the wild type (34). Unfortunately, the possible causative role suggested for this variant (34), has not been confirmed in our series of 33 unrelated kindreds.

Among other candidate genes, some involved in thyroid morphogenesis (*FOXE1* and *TTF-1/NKX.2*), or coding for a protein implicated in the regulation of the small G-protein CDC42 (*SRGAP1*), or involved in the RNA splicing machinery *(SRRM2)* have been proposed. Still, for the *HABP2* gene recently indicated as tumor suppressor, and for the *MAP2K5* gene, encoding a protein kinase that belongs to the MAP kinase family, a possible pathogenic role has been suggested. Unfortunately, none of the variants identified in these genes has not been further validated, mainly due to their absence in series of different ethnicity (18, 20–23, 32, 33), or due to the lack of co-segregation with affected members (24–27, 31), or due to their presence even in sporadic PTCs or in healthy controls (15, 24, 26, 28–31). These shortcomings could be the consequence of poor characterization of the kindreds, to ethnic differences, or to the inclusion of families with two affected members. Indeed, the presence of only two affected members in a family does not necessarily imply the hereditary origin of a TC. It has been calculated that in the presence of two affected members, the probability for a sporadic origin is 47% and for a familial origin is 53%, rising to 99.9% in the case of three affected members (43). The possible misclassification as hereditary PTCs can be applied to our series too, since the majority of our kindreds includes just two affected members. The finding of a high prevalence of somatic mutations (mainly *BRAF*V600E, followed by mutations in *TERT* or *ret/PTC* rearrangements, and some double mutations in *TERT* and in *NRAS* or *BRAF*), mainly in families with only 2 affected members, could favor the sporadic origin of the PTCs present in some families, rather than their familial origin. Nevertheless, it is worth to note that we found a somatic mutation *BRA*FV600E also in the proband from a FPTC family composed by three affected members. In accordance with other Authors (2, 14, 34, 44), we can speculate that some of our FNMTC patients could have inherited a defective gene, not yet identified, which predispose the genome to the acquisition of somatic mutations in genes typically involved in thyroid tumorigenesis.

We obtained interesting data by comparing the clinico-pathological data of the affected members of our kindreds with a large consecutive series of sporadic PTCs followed at our site. We found that familial cases were more frequently multifocal, with a higher frequency of T3-T4 tumors and a higher AJCC stage, though the outcome did not differ with respect to sporadic cases. The higher aggressiveness of familial with respect to sporadic forms of PTC is debated, though most studies reported more advanced features at diagnosis in FPTCs (4–8, 43). Nevertheless, consistent with our findings, no differences in the outcome are reported in the majority of published series (2, 4, 8, 11, 43).

In conclusion, we report genetic and clinical data in a large series of FPTC kindreds. Our families are negative for variants previously reported as likely causative, namely those lying in the *HABP2*, *MAP2K5*, and *DUOX2* genes. To confirm a possible pathogenic role for these and other susceptibility genes, careful replication studies are certainly needed. Moreover, alternative mechanisms of inheritance, such as epigenetic factors, likely warrant further investigation.

The extensive review of the current knowledge on the genetic risk factors for non-syndromic FNMTCs, here reported, underlies how the management of non-syndromic FNMTCs remains mainly clinical. FPTCs have a more aggressive clinical presentation, though, when recognized and treated appropriately, do not associate with a worst outcome with respect to sporadic cases.

## Data Availability Statement

The raw data supporting the conclusions of this article will be made available by the authors, without undue reservation.

## Ethics Statement

The studies involving human participants were reviewed and approved by Ethical Committee of the Istituto Auxologico Italiano IRCCS. The patients/participants provided their written informed consent to participate in this study.

## Author Contributions

VC: she was involved in the design of the study, in the molecular analysis of the *DUOX2* gene, in the interpretation of the results and in the first compilation of the manuscript. CC: she was involved in the evaluation of clinical and histological characteristics of all patients and in the molecular characterization of tumor tissues available. OK: she was involved in the molecular analysis of the *DUOX2* gene. GP: he was involved in the molecular analysis of the *DUOX2* gene. LP: he revised the design of the study and the final version of the manuscript. LF: she designed and supervised all the experiments of the study and wrote the paper. All authors contributed to the article and approved the submitted version.

## Funding

This work was partially supported by the Ministero dell’Istruzione, dell’Università e della Ricerca (PRIN 2017YTWKWH).

## Conflict of Interest

The authors declare that the research was conducted in the absence of any commercial or financial relationships that could be construed as a potential conflict of interest.

## References

[1] Peiling Yang S Ngeow J Familial non-medullary thyroid cancer: unraveling the genetic maze. Endocr Relat Cancer (2016) 23:R577–95. 10.1530/ERC-16-0067 27807061

[2] Moses W Weng J Kebebew E Prevalence, clinicopathologic features, and somatic genetic mutation profile in familial versus sporadic nonmedullary thyroid cancer. Thyroid (2011) 21:367–71. 10.1089/thy.2010.0256 PMC3070337 21190444

[3] Guilmette J Nosé V Hereditary and familial thyroid tumours. Histopathology (2018) 72:70–81. 10.1111/his.13373 29239041

[4] Uchino S Noguchi S Kawamoto H Yamashita H Watanabe S Yamashita H Familial nonmedullary thyroid carcinoma characterized by multifocality and a high recurrence rate in a large study population. World J Surg (2002) 26:897–902. 10.1007/s00268-002-6615-y 11965446

[5] Lee YM Yoon JH Yi O Sung TY Chung KW Kim WB Familial history of non-medullary thyroid cancer is an independent prognostic factor for tumor recurrence in younger patients with conventional papillary thyroid carcinoma. J Surg Oncol (2014) 109:168–73. 10.1002/jso.23447 24132694

[6] Tavarelli M Russo M Terranova R Scollo C Spadaro A Sapuppo G Familial non-medullary thyroid cancer represents an independent risk factor for increased cancer aggressiveness: a retrospective analysis of 74 families. Front Endocrinol (Lausanne) (2015) 6:117. 10.3389/fendo.2015.00117 26284028 PMC4522563

[7] Jiwang L Zhendong L Shuchun L Bo B Yanguo L Clinicopathologic characteristics of familial versus sporadic papillary thyroid carcinoma. Acta Otorhinolaryngol Ital (2015) 35:234–42. PMC4731882 26824209

[8] Sezer H Demirkol MO Yazici D Kapran Y Alagöl MF The clinicopathologic characteristics of familial and sporadic papillary thyroid carcinoma in Turkish patients. Turk J Med Sci (2020) 250:360–8. 10.3906/sag-1907-94 PMC7164763 31999407

[9] Capezzone M Marchisotta S Cantara S Busonero G Brilli L Pazaitou-Panayiotou K Familial non-medullary thyroid carcinoma displays the features of clinical anticipation suggestive of a distinct biological entity. Endocr Relat Cancer (2008) 15:1075–81. 10.1677/ERC-08-0080 18832444

[10] Loh KC Familial nonmedullary thyroid carcinoma: a meta-review of case series. Thyroid (1997) 7:107–13. 10.1089/thy.1997.7.107 9086578

[11] Pinto AE Silva GL Henrique R Menezes FD Teixeira MR Leite V Familial vs sporadic papillary thyroid carcinoma: a matched-case comparative study showing similar clinical/prognostic behaviour. Eur J Endocrinol (2013) 170:321–7. 10.1530/EJE-13-0865 24272198

[12] Ngan ES Lang BH Liu T Shum CK So MT Lau DK A germline mutation (A339V) in thyroid transcription factor-1 (TITF-1/NKX2.1) in patients with multinodular goiter and papillary thyroid carcinoma. J Natl Cancer Inst (2009) 101:162–75. 10.1093/jnci/djn471 19176457

[13] He H Bronisz A Liyanarachchi S Nagy R Li W Huang Y SRGAP1 is a candidate gene for papillary thyroid carcinoma susceptibility. J Clin Endocrinol Metab (2013) 98:E973–80. 10.1210/jc.2012-3823 PMC3644596 23539728

[14] Pereira JS da Silva JG Tomaz RA Pinto AE Bugalho MJ Leite V Identification of a novel germline foxe1 variant in patients with familial non-medullary thyroid carcinoma (FNMTC). Endocrine (2015) 49:204–14. 10.1007/s12020-014-0470-0 25381600

[15] Tomsic J He H Akagi K Liyanarachchi S Pan Q Bertani B A germline mutation in SRRM2, a splicing factor gene, is implicated in papillary thyroid carcinoma predisposition. Sci Rep (2015) 5:10566. 10.1038/srep10566 26135620 PMC4488885

[16] Gara SK Jia L Merino MJ Agarwal SK Zhang L Cam M Germline HABP2 mutation causing familial nonmedullary thyroid cancer. N Engl J Med (2015) 373:448–55. 10.1056/NEJMoa1502449 PMC4562406 26222560

[17] Ye F Gao H Xiao L Zuo Z Liu Y Zhao Q Whole exome and target sequencing identifies MAP2K5 as novel susceptibility gene for familial non-medullary thyroid carcinoma. Int. J Cancer (2019) 144:1321–30. 10.1002/ijc.318252 30132833

[18] Cantara S Capuano S Formichi C Pisu M Capezzone M Pacini F Lack of germline A339V mutation in thyroid transcription factor-1 (TITF-1/NKX2.1) gene in familial papillary thyroid cancer. Thyroid Res (2010) 3:4. 10.1186/1756-6614-3-4 20701785 PMC2930630

[19] Zhang T Xing M HABP2 G534E mutation in familial nonmedullary thyroid cancer. J Natl Cancer Inst (2016) 108:djv415. 10.1093/jnci/djv415 PMC4909127 26832773

[20] Zhao X Li X Zhang X HABP2 mutation and nonmedullary thyroid cancer. N Engl J Med (2015) 373:2084. 10.1056/NEJMc1511631 26581003

[21] Alzahrani AS Murugan AK Qasem E Al-Hindi H HABP2 gene mutations do not cause familial or sporadic non-medullary thyroid cancer in a highly inbred middle eastern population. Thyroid (2016) 26:667–71. 10.1089/thy.2015.0537 26906432

[22] Cantara S Marzocchi C Castagna MG Pacini F HABP2 G534E variation in familial non-medullary thyroid cancer: an Italian series. J Endocrinol Invest (2017) 40:557–60. 10.1007/s40618-016-0583-9 27873212

[23] de Mello LEB Araujo AN Alves CX de Paiva FJP Brandão-Neto J Cerutti JM The G534E variant in HABP2 is not associated with increased risk of familial nonmedullary thyroid cancer in Brazilian Kindreds. Clin Endocrinol (Oxf) (2017) 87:113–4. 10.1111/cen.13352 28418605

[24] Tomsic J Fultz R Liyanarachchi S He H Senter L de la Chapelle A HABP2 G534E variant in papillary thyroid carcinoma. PLoS One (2016) 11:e0146315. 10.1371/journal.pone.0146315 26745718 PMC4706330

[25] Ruiz-Ferrer M Fernández RM Navarro E Antiñolo G Borrego S G534E variant in HABP2 and nonmedullary thyroid cancer. Thyroid (2016) 26:987–8. 10.1089/thy.2016.0193 PMC4939372 27245704

[26] Weeks AL Wilson SG Ward L Goldblatt J Hui J Walsh JP HABP2 germline variants are uncommon in familial nonmedullary thyroid cancer. BMC Med Genet (2016) 17:60. 10.1186/s12881-016-0323-1 27530615 PMC4988026

[27] Colombo C Muzza M Proverbio MC Ercoli G Perrino M Cirello V Segregation and expression analyses of hyaluronan-binding protein 2 (HABP2): insights from a large series of familial non-medullary thyroid cancers and literature review. Clin Endocrinol (Oxf) (2017) 86:837–44. 10.1111/cen.13316 28222214

[28] Sahasrabudhe R Stultz J Williamson J Lott P Estrada A Bohorquez M The HABP2 G534E variant is an unlikely cause of familial non-medullary thyroid cancer. J Clin Endocrinol Metab (2016) 10:1098–103. 10.1210/jc.2015-3928 PMC4803181 26691890

[29] Bohórquez ME Estrada AP Stultz J Sahasrabudhe R Williamson J Lott P The HABP2 G534E polymorphism does not increase nonmedullary thyroid cancer risk in Hispanics. Endocr Connect (2016) 5:123–7. 10.1530/EC-16-0017 PMC5002962 27097599

[30] Kowalik A Gąsior-Perczak D Gromek M Siołek M Walczyk A Pałyga I The p.G534E variant of HABP2 is not associated with sporadic papillary thyroid carcinoma in a Polish population. Oncotarget (2017) 8:58304–8. 10.18632/oncotarget.16870 PMC5601653 28938557

[31] Kern B Coppin L Romanet P Crépin M Szuster I Renaud F Multiple HABP2 variants in familial papillary thyroid carcinoma: Contribution of a group of “thyroid-checked” controls. Eur J Med Genet (2017) 60:178–84. 10.1016/j.ejmg.2017.01.001 28089742

[32] Colombo C Fugazzola L Muzza M Proverbio MC Cirello V Letter regarding the article: “Multiple HABP2 variants in familial papillary thyroid carcinoma: Contribution of a group of “thyroid-checked” controls” by Kern et al. Eur J Med Genet (2018) 61:104–5. 10.1016/j.ejmg.2017.07.012 28779995

[33] Cirello V Colombo C Persani L Fugazzola L Absence of the MAP2K5 germline variants c.G961A and c.T1100C in a wide series of familial nonmedullary thyroid carcinoma Italian families. Int J Cancer (2019) 145:600. 10.1002/ijc.32244 30828786

[34] Bann DV Jin Q Sheldon KE Houser KR Nguyen L Warrick JI Genetic variants implicate dual oxidase-2 in familial and sporadic nonmedullary thyroid cancer. Cancer Res (2019) 79:5490–9. 10.1158/0008-5472.CAN-19-0721 31501191

[35] Muzza M Fugazzola L Disorders of H2O2 generation. Best Pract Res Clin Endocrinol Metab (2017) 31:225–40. 10.1016/j.beem.2017.04.006 28648510

[36] Amin MB Edge S Greene F Byrd DR Brookland RK Washington MK AJCC Cancer Staging Manual. 8th New York: Springer (2017).

[37] Haugen BR Alexander EK Bible KC Doherty GM Mandel SJ Nikiforov YE 2015 American Thyroid Association management guidelines for adult patients with thyroid nodules and differentiated thyroid cancer: The American Thyroid Association guidelines task force on thyroid nodules and differentiated thyroid cancer. Thyroid (2016) 26:1–133. 10.1089/thy.2015.0020 26462967 PMC4739132

[38] Pacini F Basolo F Bellantone R Boni G Cannizzaro MA De Palma M Italian consensus on diagnosis and treatment of differentiated thyroid cancer: joint statements of six Italian societies. J Endocrinol Invest (2018) 41:849–76. 10.1007/s40618-018-0884-2 29729004

[39] Muzza M Rabbiosi S Vigone MC Zamproni I Cirello V Maffini MA The clinical and molecular characterization of patients with dyshormonogenic congenital hypothyroidism reveals specific diagnostic clues for DUOX2 defects. J Clin Endocrinol Metab (2014) 99:E544–53. 10.1210/jc.2013-3618 24423310

[40] Pesenti C Muzza M Colombo C Proverbio MC Farè C Ferrero S MassARRAY-based simultaneous detection of hotspot somatic mutations and recurrent fusion genes in papillary thyroid carcinoma: the PTC-MA assay. Endocrine (2018) 61:36–41. 10.1007/s12020-017-1483-2 29214440 PMC5997117

[41] Colombo C Muzza M Proverbio MC Tosi D Soranna D Pesenti C Impact of mutation density and heterogeneity on papillary thyroid cancer clinical features and remission probability. Thyroid (2019) 29:237–51. 10.1089/thy.2018.0339 30501571

[42] Ameziane-El-Hassani R Schlumberger M Dupuy C NADPH oxidases: new actors in thyroid cancer? Nat Rev Endocrinol (2016) 12:485–94. 10.1038/nrendo.2016.64 27174022

[43] Triponez F Wong M Sturgeon C Caron N Ginzinger DG Segal MR Does familial non-medullary thyroid cancer adversely affect survival? World J Surg (2006) 30:787–93. 10.1007/s00268-005-0398-x 16479341

[44] Cavaco BM Batista PF Martins C Banito A do Rosário F Limbert E Familial non-medullary thyroid carcinoma (FNMTC): analysis of fPTC/PRN, NMTC1, MNG1 and TCO susceptibility loci and identification of somatic BRAF and RAS mutations. Endocr Relat Cancer (2008) 15:207–15. 10.1677/ERC-07-0214 18310288

